# An increased tendency in fibrinogen activity and its association with a hypo-fibrinolytic state in early stages after injury in patients without acute traumatic coagulopathy (ATC)

**DOI:** 10.1007/s11239-018-1642-1

**Published:** 2018-03-21

**Authors:** S. He, M. Blombäck, F. Boström, H. Wallen, J. Svensson, A. Östlund

**Affiliations:** 1 Department of Clinical Sciences, Danderyd Hospital, Karolinska Institutet, Building 8-9, 6th floor, 18288 Stockholm, Sweden; 2 Department of Molecular Medicine and Surgery, Karolinska Institutet, Stockholm, Sweden; 3 Department of Clinical Immunology and Transfusion Medicine, Karolinska Institutet, Stockholm, Sweden; 4 Perioperative Medicine and Intensive Care, Karolinska University Hospital, Karolinska Institutet, Stockholm, Sweden; 5 Department of Physiology and Pharmacology, Section for Anesthesiology and Intensive Care Medicine, Karolinska Institutet, Stockholm, Sweden

**Keywords:** Acute traumatic coagulopathy, Thrombin generation, Fibrinogen, Coagulation, Clot proteolysis

## Abstract

Acute traumatic coagulopathy (ATC) diagnosed by prolongation of APTT and/or PT/INR involves alterations in platelet activity, coagulation and fibrinolysis. However, data showing the haemostatic situation in injured patients without ATC are scarce. To assess whether haemostatic impairment is also present in injured patients without ATC, ten injured patients without ATC and ten normal individuals were examined. The patients were sampled on arrival at the emergency department 0, 2, 12 h after surgical or other intervention. Thrombin generation, fibrin formation and fibrin proteolysis were determined via several laboratory methods, using tissue factor as the coagulation trigger. Thrombograms demonstrated that trauma accelerated both thrombin generation and decay. In the presence of unaffected peak thrombin levels, these two contradictory effects cancelled each other out, leading to the global endogenous thrombin potential (ETP) remaining normal. Under the mediation of normal ETP, fibrin network permeability (Ks) kept the reference levels in the two groups of subjects. Fibrinogen (FBG) activity (Clauss) rose with time from 0 to 2 h and 12 h, which significantly slowed down Clot Lysis Potential as determined by an in vitro method with exogenous t-PA. Summary: the main haemostatic impairment in the present patients concerned an increased tendency in FBG activity. Since an increase in FBG is a biomarker of acute inflammation and also predicts greater fibrin production which down-regulates fibrinolysis, we suggest that during early stages after injury, patients without ATC may suffer from worsening inflammation and confront enhancement of thrombosis risk due to dysfunction of fibrinolysis.

## Introduction

Major traumas bring a variety of disorders to haemostatic function, thereby increasing the risk of haemorrhage soon after injury, and/or thrombosis later [1, 2]. When trauma patients arrive at emergency departments, values of INR > 1.5 or APTT > 1.5 times normal are used to identify early coagulation deficiencies, termed acute traumatic coagulopathy (ATC). ATC has adverse effects on clinical outcomes and mortality [3, 4].

Developed knowledge of the coagulation cascade [5] has prompted clinicians and researchers into consideration that APTT and/or PT/INR (as signals of time-to-start of detectable fibrin) cannot fully characterize dynamic impairments in the global haemostatic system caused by trauma and may thus result in inadequate guidance in emergency management. Therefore, in recent years, numerous investigations have been focused on assessment of how ATC alters the overall haemostasis potential, including platelet activity, coagulation and fibrinolysis. Accumulated findings support the idea that the pathogenic mechanisms underlying ATC involve interplay between platelet dysfunction, acquired fibrinogen insufficiency, enhancements in endothelial activation, the inflammation response, endogenous anticoagulation and clot lysis by plasmin, etc. However, data showing the haemostatic situation in injured patients without ATC are scarce in the literature [6].

The present study involved 10 patients who experienced major injuries after traumatic accidents. When they arrived at the emergency department, APTT values were 24.0–30.0 s, while those of INR were ≤ 1.5. Multiple laboratory tools known to be useful for scanning the global effects of coagulation and fibrinolysis were employed, aiming to test whether coagulation and fibrinolysis act abnormally to a certain extent in injured patients without ATC. Because only platelet-poor plasma samples were available in our study, no assays of platelet activity were carried out.

## Materials and methods

### Clinical characteristics of the patients

The study involved 10 patients (patient group) with major trauma. They were aged 25–56 years (median 35 years) and there were two women and eight men (Table 1). They all were admitted to the Intensive Care Unit at Karolinska University Hospital, Solna; the median (range) time between injury and arrival at the trauma unit were 44 (7–66) min. All study procedures with the trauma patients’ plasma were conducted after approval from the regional ethics committee at Karolinska University Hospital.

**Table 1:** Characteristics of the injured patients without acute traumatic coagulopathy

Patient no.	Age	Trauma	Injuries	Acute intervention/surgery	Transfusion	Synthetic colloids
1	27	Motor bike acc.	Rib fracture, lung and liver contusion	No acute intervention	N	N
2	31	Motor bike acc.	Open arm fracture	Ext. fixation of fracture	N	N
3	56	Motor bike acc.	Rib and scapular fractures, liver contusion	Thoracic drain	N	N
4	25	Crush injury	Shoulder injury	Reposition	N	N
5	55	Car acc.	Facial and fibula fracture	No acute intervention	N	N
6	35	Crush injury	Pelvic fracture, liver contusion, aortic dissect	Stenting aorta + laparotomy	4 U RBC + 1 U plasma	Y
7	45	Crush injury	Sternal fracture, liver laceration	Laparotomy	N	Y
8	34	Car acc.	Femur facture	Traction femur	N	N
9	31	Crush injury	Open tibial facture, crush injury leg	Ext. fix. femur revision	4 U RBC	Y
10	42	Crush injury	Pelvic, rib and femur fracture, lung contusion	Bilat. ext. fixation of femur	4 U RBC + 4 U plasma	Y

*Acc*. accident, *U* unit, *RBC* red blood cell, *N* no, *Y* yes

In the patient group, 20 ml of whole blood was taken at each of three time-points: 0 h = on arrival at the emergency department, 2 and 12 h = 2 and 24 h after termination of surgical operation or other forms of intervention, repectively. Via an antecubital vein, whole blood was collected into evacuated tubes containing 0.13 M citrate (one part trisodium citrate to nine parts of blood) with use of minimal stasis. The citrated blood samples were centrifuged within 30 min (2000×*g*; 20 min; room temperature). The platelet-poor plasma obtained was stored in small aliquots at − 80 °C.

All patients (Table 1) received Ringer’s acetate (1000–3000 mL depending on bleeding) between the sampling times of 0 and 2 h. Patients 1–5 and 8 were relatively stable and did not require transfusion. Red blood cell concentrate was given to patients 6, 9 and 10 because of bleeding; two of them (6 and 10) also received plasma. Moreover, a synthetic colloid (Voluven, Fresenius Kabi) was given to patients 6, 7, 9 and 10 as a volume expander during operations. Patient 7 also received low-molecular-weight heparin (Klexane, 40 mg) at the time-points of 2 and 12 h for thrombosis prophylaxis. None of the patients was on treatment with any anti-fibrinolysis agent. All the patients left the hospital alive.

### Commercial plasma samples

A normal citrated platelet-poor plasma pool (NPP, used as control in all tests) was provided by Precision Biologic, Dartmouth, Canada.

Ten single-donor plasma samples (citrated platelet-poor) were collected from 10 normal individuals (“reference group”); females three, males seven; mean age 41 years (median 41 years). All these individuals had normal APTT, INR, and fibrinogen activity as reported by the supplier, George King Bio-Medical, Overland Park, KS, USA.

### Special reagents

One vial of Innovin® (recombinant human TF: rTF) from Siemens Healthcare Diagnostics, Marburg, Germany was reconstituted with 10 mL distilled H_2_O (theoretical concentration 6000 pmol/L according to the manufacturer) and then kept at − 20 °C in small aliquots. A purified phospholipid mixture (PPL, 500 µM) from Rossix, Mölndal, Sweden, was kept at 4–8 °C. Recombinant tissue-type plasminogen activator (rt-PA; Actilyse®) from Boehringer Ingelheim, Germany, was prepared (1 mg/mL) with the manufacturer’s solvent and kept in small aliquots at − 70 °C.

### Assays performed in the routine laboratory

In the routine laboratory at Karolinska University Hospital (Solna), APTT, INR, haemoglobin, platelet count and fibrinogen activity (Clauss; normal range 2.0–4.2 g/L) were assayed.

### Assays performed in our laboratory

**Fig. 1:**
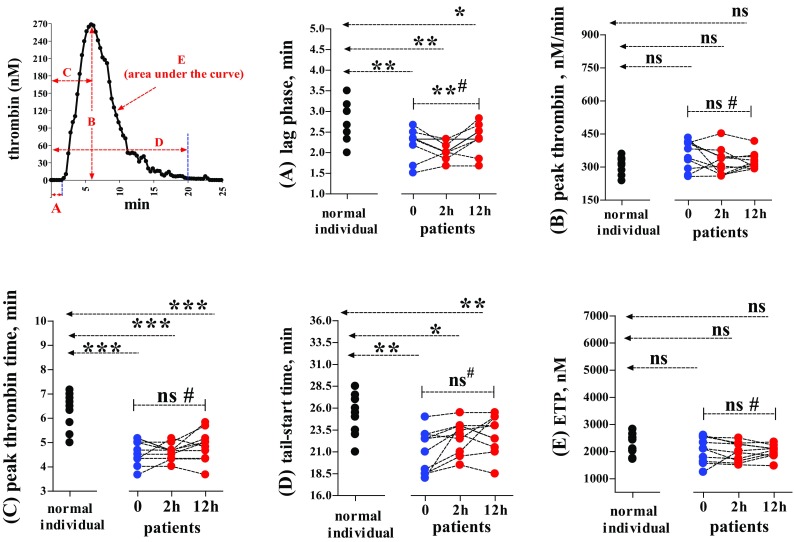
Variables of CAT measurement in the trauma patients and the normal individuals (reference group). Normal: normal individuals (n = 10). Patients: trauma patients (n = 10) sampled at time point 0 (arrival at the emergency department), and time points 2 and 12 h (2 and 12 h after termination of surgery or other forms of intervention). CAT variables: **A** lag phase = time-to-start of detectable thrombin generated, **B** peak thrombin = maximum concentration of thrombin generated, **C** peak thrombin time = time taken to reach “peak thrombin”, **D** tail-start time = time taken to observe the full deterioration of generated thrombin, and **E** ETP endogenous thrombin potential, calculated as the area under the curve during the whole registration time. Dotted line with an arrow = Variables from the normal individual group compared with those from the patients at 0, 2 or 12 h were analysed by using Mann Whitney test. # and dotted line with two bars = Variables from the same patients between different time points (0, 2, and 12 h) were compared by using Repeated measures ANOVA test. ns = *p* > 0.05, *p < 0.05, **p < 0.01 and ***p < 0.0001. In CAT assays, plasma was clotted by adding a coagulation trigger solution including recombinant tissue factor (rTF, 5 pM), purified phospholipids (PPLs, 4 µM) and fluorogenic substrate together with CaCl_2_, i.e., FluCa (20 mM); plasma volume versus volume of reagent solution was 80 versus 40 µL (2:1). See “Materials and methods” for more information

Calibrated automated thrombogram (CAT) measurement for determining endogenous thrombin generation was carried out according to the instructions of the manufacturer (software version 2007, Thrombinoscope, BV, Maastricht, the Netherlands) [7], though the coagulation trigger was prepared by ourselves. Briefly, Tris buffer was prepared by dissolving 50 mM Trizma base (Sigma-Aldrich, St. Louis, Missouri, US) and 0.1 M NaCl in 1 L distilled water; pH 7.4 was adjusted with HCl. In each well of a microplate, 80 µL of a plasma sample was mixed with a solution (20 µL) containing rTF and PPL in the Tris buffer. Then, 20 µL of a commercial fluorogenic substrate (which can specifically be cleaved by thrombin) in combination with CaCl_2_ was added to initiate coagulation. In the reaction mixture, final concentrations were rTF 5 pmol/L, PPL 4 µmol/L and CaCl_2_ 20 mmol/L, while plasma dilution by the reagent solutions was 80:(20 + 20), i.e., 2:1. The fluorescence in the sample was recorded every 30 s for 60 min using a fluorometer (Fluoroscan Ascent, Thermo Scientific, Vanta, Finland), to set up a curve showing generated thrombin quantity against time. The different variables obtained were “lag phase” (time-to-start of detectable thrombin), “peak thrombin” (maximum concentration of thrombin formed), “peak thrombin time” (time taken to reach “peak thrombin”), “tail-start time” (time taken to observe the full deterioration of generated thrombin) and the global “endogenous thrombin potential” (ETP, area under the curve during the whole registration period) (Fig. 1).

Fibrin network permeability was determined according to the approach originally described by Blombäck and Okada [8] and later modified by our group [9]. In short, fibrin clots were prepared, by mixing 160 µL plasma with 80 µL of a coagulation trigger solution (rTF, PPL and CaCl_2_ in Tris buffer). The final concentrations of the different reagents (rTF, 5 pmol/L, PPL, 4 µmol/L and CaCl_2_, 20 mmol/L) and plasma dilution by the reagent solutions (160:80 = 2:1) were identical to those in the CAT assay. Next, 200 µL of the sample mixture was immediately added to a plastic cylinder made of a Combitips Plus tip (0.5 mL Eppendorf, Hamburg, Germany). The fibrin gel was incubated overnight for stabilization in a moisture chamber at room temperature. The stabilized fibrin network was then percolated with Tris buffer for 40 min to fully wash soluble impurities away. The same percolation procedure was then repeated once more for 60 min and the volume of the eluted buffer was measured. The permeability coefficient (Ks) was calculated by using the formula:

$${\text{Ks }}( \times {10^{ - 9}},{\text{c}}{{\text{m}}^2})={\text{Q}} \times {\text{L}} \times \eta /{\text{t}} \times {\text{A}} \times \Delta {\text{P}}$$

where Q (cm^3^) = the flow rate at time t (s), L (cm) = the length of the fibrin gel, η (dyne × s/cm^2^) = the viscosity of the liquid, A (cm^2^) = the cross-sectional area and ∆p (dyne/cm^2^) = the hydrostatic pressure. In each run of the fibrin network permeability assay, one fibrin clot from the NPP sample was examined in parallel. The Ks value obtained from NPP was used as a control level, set at 100%. Fibrin network permeability measured in the subjects’ samples was normalized against the result in the NPP, reported as “% of control”.

To assess Clot Lysis Property (CLP), 160 µL plasma was mixed with 40 µL rt-PA solution in each well of a microtiter plate (Thermo, MA, US), followed by shaking at 37 °C for 1 min. After adding 40 µL of a coagulation trigger solution containing rTF, PPL and CaCl_2_, fibrin optical density (OD) was kinetically recorded for 5 h, i.e., 18,000 s (405 nm; 24 °C) by using a spectrophotometer (Tecan, Gröding, Austria). The final concentrations of the different reagents (rTF, PPL and CaCl_2_) and the plasma dilution by the reagent solutions [160:(40 + 40) = 2:1] were identical to those in the CAT and Ks assays, above, while the final concentration of rt-PA was 195 ng/mL. This rt-PA concentration is at least tenfold higher than the physiological level but lower than the mean therapeutic level during thrombolysis performed clinically [10]. According to the obtained raw data (OD values against reading time), a CLP variable was defined via two steps: Step I, determining maximum OD value (A), time to maximum OD value (B) and OD value at B plus1800s (C); Step II, CLP = (A – C)/A × 100%. Thus, CLP reflects the percentage of maximum fibrin lysed up to the 1800th second after the maximum amplitude of fibrin quantity.

### Statistical methods

All data were statistically analysed on a PC system using GraphPad Prism software, version 4 (Graph Pad, San Diego, Cal., USA). Variables from the normal individual group versus those from the patients at 0, 2 or 12 h (unpaired data) were compared by using Mann Whitney test. Variables from the same patient between different time points of 0, 2 and 12 h (paired data) were compared by using the repeated measure ANOVA test. Spearman’s rank correlation coefficient (r_s_) was determined to assess the dependence between two variables. A value of *p* < 0.05 was considered to be significant.

## Results

Assayed at the time-point of 0 h, APTT (median 28.5 s; range 24.0–30.0; normal level 20–30), INR (median 1.1; range 0.9–1.2; normal level < 1.2), haemoglobin (median 144.5 g/L; range 124–165; normal level 117–170) and platelet count (median 231 × 10^9^/L; range 148–299; normal level 145–387) revealed normal outcomes.

Compared with plasma from the normal individuals (unpaired), the patients’ samples at all three time-points (0, 2 and 12 h) showed significant decreases in both “lag phase” and “peak thrombin time”; yet, there was no significant change in “peak thrombin” or ETP (Fig. 1A, B, C, E). Between the different time-points 0, 2 and 12 h, significant variations only appeared in “lag phase” but not in “peak thrombin” and “thrombin peak time” (paired). As regards “tail-start time” (Fig. 1D), significant shortening was seen at 0, 2 or 12 h versus values in the reference group (unpaired). Between 0, 2 and12 h, this variable did not differ significantly (paired).

**Fig. 2:**
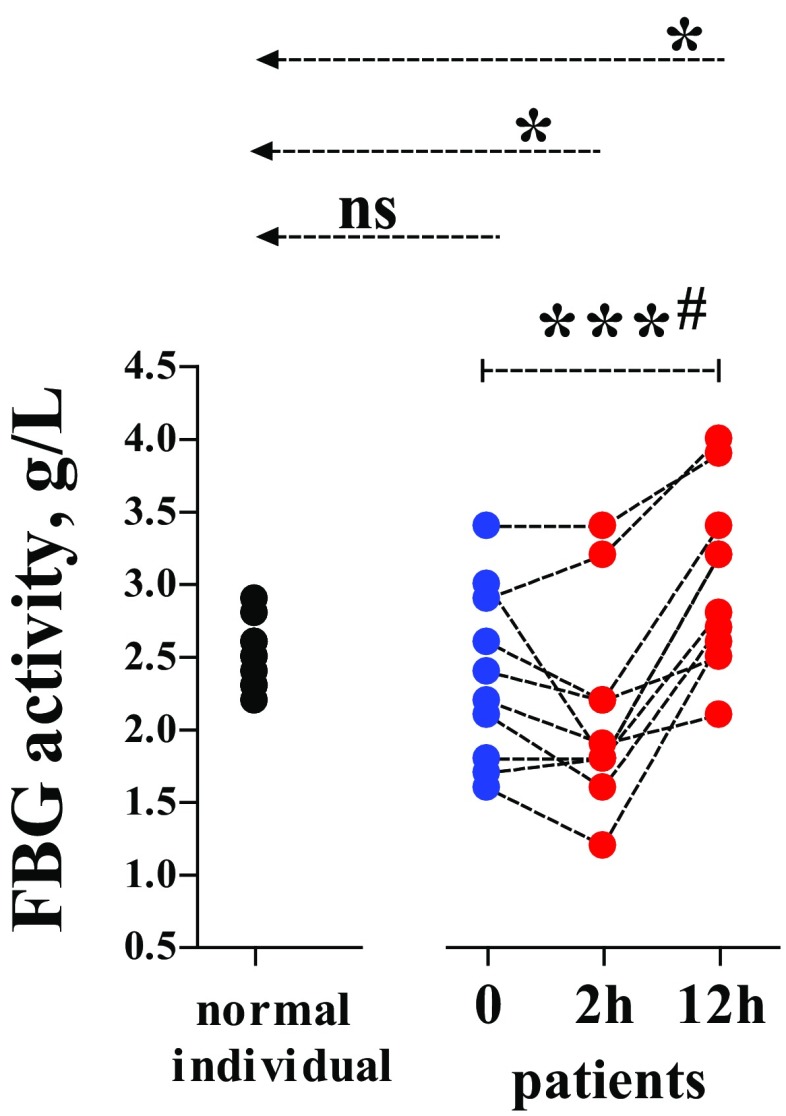
Fibrinogen (FBG) activity in the trauma patients and the normal individuals (reference group). FBG activity was determined by using the Clauss method. See Fig. 1 or “Materials and methods” for more information

In the whole observation period, FBG activity remained within the reference range (2.0–4.2 g/L). However, values of this variable became significantly higher at 2 or 12 h when compared with the healthy individual group (unpaired). The variations between the different time-points were also significant (paired) (Fig. 2).

**Fig. 3:**
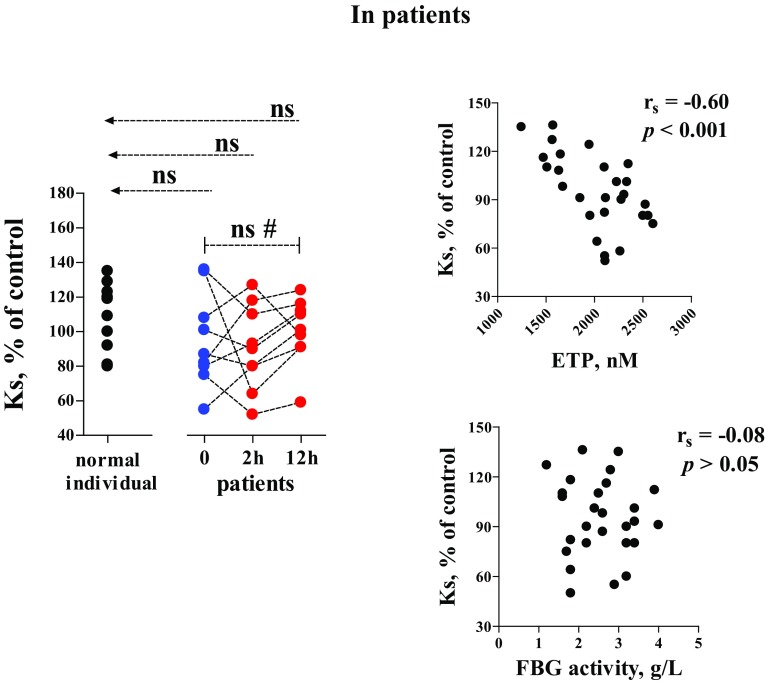
Fibrin network permeability (Ks) and its correlation to values of ETP and FBG activity. In the Ks assays, clots were prepared by mixing plasma with the coagulation trigger solution at concentrations and plasma dilution degree identical to those in the CAT assay (though CaCl_2_ was used instead of FluCa). r_s_ = Spearman’s correlation coefficient. See Figs. 1 and 2 or “Materials and methods” for more information

In the patient group, fibrin network permeability (Ks) did not differ significantly when comparisons were made between any time-point and the reference group (unpaired), or between 0, 2 and 12 h (paired) (Fig. 3, left). Using all the values of ETP, Ks and FBG obtained from the patient samples, Ks levels were correlated significantly with ETP but not FBG activity, as tested by Spearman analysis (Fig. 3, right).

Patient 7 who received low-molecular-weight heparin (Klexane, 40 mg) at the 2 and 12 h for thrombosis prophylaxis. In his samples, the CAT variables did not reveal over decline in the thrombin generation (data not shown).

**Fig. 4:**
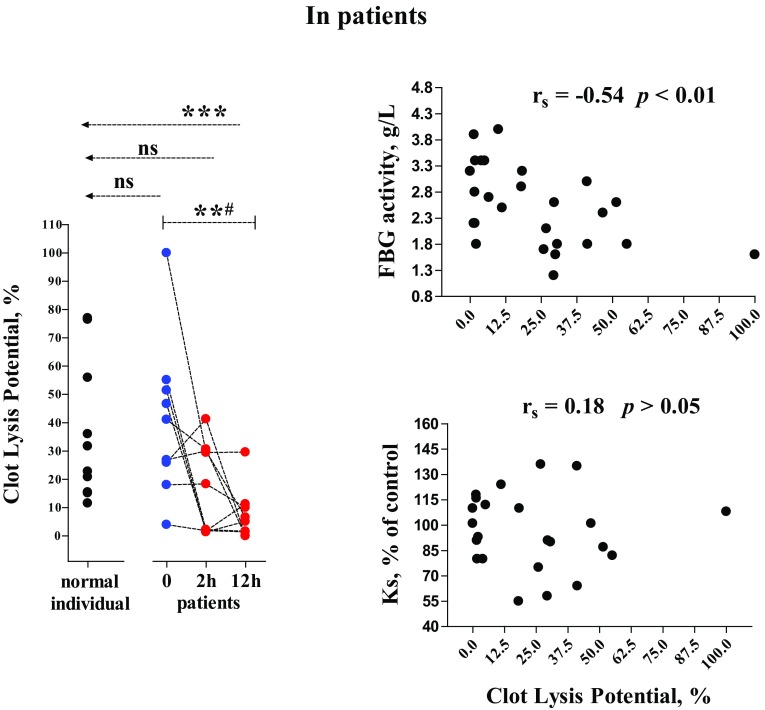
Clot Lysis property and its correlation to FBG activity or to fibrin network permeability. The laboratory and statistical methods are described above (Figs. 1, 2, 3); for more information, see “Materials and methods”

For Clot Lysis Property (CLP), there was no significant difference in the patient samples at 0 or 2 h versus the reference group (unpaired) (Fig. 4, left). However, this variable depleted significantly at 12 h after operations/other acute interventions. The differences in CLP were significant between the three time-points (paired). All the values of CLP, Ks or FBG obtained from the cases were included in the Spearman analysis, we found that CLP significantly correlated with levels of FBG (Fig. 4, right, upper) but not with Ks (Fig. 4, right, lower).

## Discussion

### Diagnosis of the patients

In view of normal levels in APTT, INR, haemoglobin and platelet count detected at the time point of 0 h, we categorize the patients as injured cases without ATC (1–4); among them, no plasma dilution was present. Three of ten patients suffered from major bleeding at 2 and 12 h, which may theoretically lead to further impairments in the haemostatic system. However, as no additional abnormality found in the clinical situation, we may consider that the transfusion of plasma and blood-cells in apposite volumes had most likely compensated the blood loss and thus prevented happening of supplementary coagulopathy.

### Unification of the methods

An identical coagulation trigger solution 5 pM rTF in combination with 4.4 µM PPL and 1 mM CaCl_2_, as well as an identical plasma dilution degree (2:1), were used in the various analyses (CAT, fibrin network permeability and CLP). Fibrin clot formation in the different analyses was hence based on the same coagulation mechanism. This allows us to consider that the following discussions on interactions of thrombin generation potential, fibrin network permeability and clot stability against proteolysis go along with the physiological mechanisms.

### Thrombin generation

Via monitoring cleavage of a fluorogenic substrate after stimulation with rTF etc., CAT measurement in plasma samples is highly sensitive as regards assessment of thrombin generation and deterioration [7]. Even so, only a limited number of studies has involved use of this advanced laboratory tool to learn whether or not thrombin generation is altered by trauma [6, 11, 12]. Nacy et al. [6, 11, 12] found that endogenous thrombin generation potential (ETP) remained normal in injured patients without ATC, where “lag phase” was similar to normal control, while “peak thrombin” rose significantly (*p* < 0.01). Information about “peak thrombin time” or “tail-start time” was absent in their report.

In the present study, however, we have evaluated all the CAT variables in the two subject groups (Fig. 1). Median levels of “lag phase” and “peak thrombin time” were significantly shorter in the patients than in the normal individuals. That is to say, the start of thrombin generation and the appearance of the highest thrombin concentration shifted to an earlier time soon after the accidents. Since most patients did not experience the severe blood loss and over-infusion while a few patients with bleeding timely accepted transfusion (Table), Factor II concentrations may not have been altered critically and so maintained the original levels of “peak thrombin”. Moreover, “tail-start time” reflects the rapidity of thrombin decay in association with the anti-thrombotic functions of the protein C pathway [13], and maybe antithrombin. Damaged endotheliumin in trauma could release thrombomodulin, which facilitates protein C activation, with its inhibitory effects on Factor V and Factor VIII [3, 14]. According to this concept, the significant shortening of “tail-start time” in the patients’ plasma may be thought as a consequence of endothelial damage by trauma. Thus, traumatic injuries may accelerate both thrombin generation and its deterioration. In the presence of unaffected levels of “peak thrombin”, these contradictory effects cancel each other out, leading to comparable total amounts of generated thrombin (shown as ETP) in the two subject groups.

### Fibrinogen activity

Since depletion of FBG by way of severe bleeding and plasma dilution has been viewed as the chief pathogenetic mechanism of ATC [3, 4], we were allowed to assume that injured patients without ATC possess normal plasma levels of FBG. As expected, FBG activities in our patients were analogous to those in the reference group (Fig. 2). However, FBG activity in the patient group displayed an increasing tendency along with the time advance from 0 to 12 h. This should indicate that the injured patients—who though having avoided ATC—still suffered worsening inflammation, because elevation in FBG activity is regarded as a biomarker of acute inflammation in many pathological conditions [15]. Unfortunately, we had no samples available for analysing other inflammation markers which perhaps would verify this inference.

### Fibrin network permeability

According to the comparison between the patients and normal individuals, and between time-points 0 and 2/12 h (Fig. 3, left), we realized that traumatic injuries did not modify fibrin network permeability significantly. This observation may account for the fact that the two subject groups owned analogous levels of global thrombin generation potential—ETP (Fig. 1E), which permitted variations of Ks to a similar extent (Fig. 3, right).

The lack of a significant correlation between Ks and FBG (Fig. 3, right, lower) is a great challenge to the traditional concept i.e., plasma concentrations of FBG down-regulated fibrin network permeability [16, 17]. Actually, the published studies cannot reflect the actual situation in vivo, since exogenous thrombin was used to activate coagulation which overwhelms endogenous thrombin generation and force fibrin formation into critical dependence upon the FBG quantity and/or FBG clotting properties. In the present study, we employed a physiological coagulation trigger—rTF to elicit the activation of prothrombin [18]; the endogenously induced thrombin acted as the chief role to control the pore sizes of the fibrin network and the related liquid permeability. As regards FBG, its effect on fibrin formation may only give rise to an increase in fibrin amount and fibrin fibre thickness [19].

### Clot lysis by plasmin

Fibrinolysis is a compensatory effect in coagulation that keeps vascular patency via breaking down excessive fibrin clots. Injured patients with ATC frequently have hyper-fibrinolysis as a complication—a vital origin of haemorrhage [3, 4]. A hypo-fibrinolytic state can also be observed in trauma patients, a condition called “fibrinolysis shutdown” [20, 21]. “Fibrinolysis shutdown” is known to be fundamental in the occurrence of microvascular thrombosis and organ failure post trauma.

Assay of clot lysis time (CLT) has been extensively performed to identify the fibrinolysis rate [9, 19]. However, in our preliminary study, we found that the CLT method is not suitable for examining the patients with hypo-fibrinolysis, where CLT i.e., the time point to obtain 50% decrease of maximum OD is later than 5 h (the limited assay period). We therefore designed another approach, i.e., CLP as introduced above. Compared with the reference group, CLP values were similar between the time points 0 and 2 h in the case group. However, the patient samples obtained at 12 h showed significantly lower values of CLP than in the normal individuals and in the patients sampled at 0 and 2 h (Fig. 4, left). We are not sure whether these assay results replicate “fibrinolysis shutdown” which is primarily assessed by the outcome of thrombelastography in other trauma patients [20, 21]; but a proposed existence of a hypo-fibrinolytic state in injured patients without ATC should be reasonable.

For the moderate significance in statistical correlation between CLP and FBG activity (Fig. 4, right, lower), an explanation may be suggested by that more functional fibrinogen molecules give rise to higher production of fibrin; proteolysis of greater amounts of fibrin is more time-consuming. In addition, data obtained from the present study are powerless to exclude the effects by certain fibrinolytic inhibitors, such as PAI-1; other authors have found elevated PAI-1levels after trauma though the exact mechanisms are not yet clear [20–22]. We would like to perform more assessments to address the above issue, and also to test our another inference, i.e., sufficient thrombin generation and increased effects of thrombomodulin in trauma patients (as mentioned above) may help activation of thrombin-activatable fibrinolysis inhibitor (TAFI), which in turn brings about a hypo-fibrinolytic state.

Fibrin network permeability is known to up-regulate transport of fibrinolytic components in blood, so thus favouring clot lysis by plasmin [23]. However, in the present study, CLP values did not show significant correlation with those of Ks (Fig. 4, right, lower). This unexpected finding may remind us of that in the injured patients without ATC, increase in FBG quantity—probably together with certain alterations in fibrinolytic components—is more potent in affecting fibrinolysis situation, rather than fibrin network permeability which kept on the reference levels (Fig. 3, left).

### Limitations of this study

One of the limitations of this study was that the sample sizes were too small to ensure the accuracy of statistical significance in the assay results. Another drawback was rooted in shortage of available samples for further investigations concerning other biomarkers of acute inflammation or endothelium damage, as well as the fibrinolysis activators/inhibitors.

## Summary

The major haemostatic impairment in the patient group concerned a tendency towards increased FBG activity. Since FBG is a biomarker of acute inflammation and also predicts greater fibrin production that then down-regulates fibrinolysis velocity, we suggest that during early stages after injury, patients without ATC may suffer from worsening inflammation and confront enhancement of thrombosis risk due to hypo-fibrinolysis.
